# COVID-19 Lockdown and the Behavior Change on Physical Exercise, Pain and Psychological Well-Being: An International Multicentric Study

**DOI:** 10.3390/ijerph18073810

**Published:** 2021-04-06

**Authors:** Anelise Sonza, Danúbia da Cunha de Sá-Caputo, Alessandro Sartorio, Sofia Tamini, Adérito Seixas, Borja Sanudo, Jessica Süßenbach, Marcello Montillo Provenza, Vinicius Layter Xavier, Redha Taiar, Mario Bernardo-Filho

**Affiliations:** 1 Programa de Pós-Graduação em Fisioterapia e Programa de Pós-Graduação em Ciências do Movimento Humano, Centro de Ciências da Saúde e do Esporte, Universidade do Estado de Santa Catarina, 88085-350 Florianópolis, Brazil; anelise.sonza@udesc.br; 2 Laboratório de Vibrações Mecânicas, Policlínica Piquet Carneiro, Instituto de Biologia Roberto Alcantara Gomes, Universidade do Estado do Rio de Janeiro, 20550-900 Rio de Janeiro, Brazil; dradanubia@gmail.com (D.d.C.d.S.-C.); bernardofilhom@gmail.com (M.B.-F.); 3 Faculdade Bezerra de Araújo, 23052-090 Rio de Janeiro, Brazil; 4 Istituto Auxologico Italiano, IRCCS, Experimental Laboratory for Auxo-endocrinological Research & Division of Auxology and Metabolic Diseases, 28824 Piancavallo (VB), Italy; sartorio@auxologico.it (A.S.); s.tamini@auxologico.it (S.T.); 5 Escola Superior de Saúde, Universidade Fernando Pessoa, 4200-253 Porto, Portugal; aderito@ufp.edu.pt; 6 Departamento de Educación Física y Deporte, Universidad de Sevilla, 41013 Seville, Spain; bsancor@us.es; 7 Institut für Bewegung, Sport und Gesundheit, Leuphana Universität Lüneburg, 78120 Lüneburg, Germany; suessenb@leuphana.de; 8 Instituto de Matematica e Estatistica, Universidade do Estado do Rio de Janeiro, 20550-900 Rio de Janeiro, Brazil; mprovenza@ime.uerj.br (M.M.P.); viniciuslx@ime.uerj.br (V.L.X.); 9 MATIM, Université de Reims Champagne Ardenne, 51100 Reims, France

**Keywords:** SARS-CoV-2, physical exercise, survey, stress, anxiety, social isolation, pain

## Abstract

Objective: To evaluate the impact of the COVID-19 pandemic and the following lockdown on physical exercise (PEx) practice, pain, and psychological well-being. Methods: A cross-sectional multicentric study was performed using a nonrandom convenience sampling from the general population (≥18 years-old) of 6 countries (Brazil, Italy, France, Portugal, Germany, and Spain) adopting social isolation (SI). The validated self-administered online survey (PEF-COVID19) was used. The tests T-test and Chi-square with Bonferroni correction were used for statistical analysis and a multivariate logistic regression model (*p* ˂ 0.05). Results: We included 3194 replies and ~80% of the respondents were in SI. Brazilian sample was highly influenced by the pandemic considering PEx practice and habits, pain, anxiety, and stress (*p* ˂ 0.05). Among the European countries, Italy presented the major changes. The model to predict the non-practice of PEx during SI showed that the variables countries, smoking, SI, and PEx level were significant predictors (*p* ˂ 0.001). Conclusion: The pandemic changed the PEx practice and habits, and the psychological well-being of populations in different manners. Countries, smoking, SI, and PEx level were predictors for the non-practice of PEx. Public health strategies are suggested to avoid sedentary lifestyles and quality of life decrease.

## 1. Introduction

Regular exercise causes a myriad of desirable effects with clear beneficial outcomes for health promotion, the treatment of diseases, and lifespan, and decreases mental health outcomes [1], such as anxiety [2] and depression [3], hence being considered as a polypill for health [4,5,6]. Physical exercise (PEx) is a type of physical activity (PA), that is planned, structured, repetitive, and favors physical fitness maintenance or development [7]; by contrast, physical inactivity represents the nonachievement of the expected actions related to PA [7,8,9]. At the same time, sedentary behavior, defined as “a distinct class of behaviors characterized by low energy expenditure” [10], equivalent to sitting or lying down [9] e.g., watching television, using a computer and smartphone, playing videogames for a prolonged time, traveling in motorized vehicles for short distances instead of walking, etc., moves in the opposite direction of the PEx and PA [11].

Physical inactivity and sedentary behaviors are favored by the measures adopted to curb the consequences of the coronavirus disease (COVID-19) [12,13,14], such as social distancing, ranging from isolation, quarantine, local confinement up to lockdown. The COVID-19 disease emerged in December 2019 in China and rapidly reached several countries [15,16]. Therefore, the outbreak of COVID-19 had a strong impact on people’s daily life, including the level of PEx and PA, because people reduced daily activities outside. As a logical consequence, this situation impacted the general health of the population and contributed to physical inactivity [13,14,17] and also to undesirable psychological conditions, such as insecurity and fear causing anxiety and stress [13,16,18], thoughts about death, feelings of helplessness and abandonment [18], especially for the elderly.

The undesirable sedentary lifestyle is associated with deleterious health outcomes leading to the development of several chronic diseases, including stroke, type 2 diabetes mellitus, coronary heart disease, obesity, and other comorbidities and premature mortality [19,20]. In a recent review, Ruegsegger and Booth (2018) [20] have pointed out that PA would be a noninvasive therapy for mental health improvements in cognition, depression, anxiety, neurodegenerative diseases (Alzheimer’s and Parkinson’s disease), and drug addiction. Consequently, physical inactivity and prolonged sedentary behavior may have prejudicial psychological [13,21,22,23] and physical health consequences [19], such as the rapid loss of muscle mass and neurodegeneration [12]. Besides, exercise interventions contribute to promote mental health [24] and lessen the symptoms of anxiety and stress [25].

The relationship between a sedentary lifestyle and the COVID-19 epidemic, classified as a pandemic by the World Health Organization (WHO) on 11 March 2020 [26], might exist since home-confinement is happening for several weeks or months. The first cases detected outside China were reported in France on 24 January 2020, immediately followed by nine other European countries (Belgium, Finland, France, Germany, Italy, Russia, Spain, Sweden, and the UK) on 21 February [27]. The first COVID-19 case in Brazil was confirmed on 26 February 2020 [28], and in Portugal, the first cases were reported on 2 March, with lockdown starting on 18 March 2020; at the beginning of March, cases were detected and confirmed in 72 countries [29]. Spain, despite the lockdown imposed on 14 March 2020, was one of the most affected countries worldwide [30].

Therefore, the evaluation of the impact on health due to social isolation is relevant to aid in the establishment of guidelines for the postpandemic period. Recently, studies reported and discussed sedentary behavior [13,14,31,32] and its mental health impact [13,16,33,34] during the COVID-19 pandemic in varied populations. For these reasons, assessing and identifying the changes in the level of PEx and other conditions related to daily life during the outbreak is relevant, considering outdoor physical activities are suspended. Inevitably, this confinement is associated with a series of changes in our daily life activities, which usually lead to sedentary behavior characterized by a decrease in physical activity. Questionnaires and surveys are important tools to gather information on individual perspectives in a population aiming for epidemiological surveys, surveys on attitudes to a health service or intervention [35,36]. Public policies involving PA are proposed in several fields, such as parks and public spaces, transportation, the health care sector and the school sector [37]; however, public policies to promote PA during a pandemic are very important, considering many policies closing or restricting access to outdoor places where people usually go to exercise [38].

Considering that COVID-19 spread all over the world, the use of the same survey in different countries would be relevant. Surveys are often used in cross-national research to understand which theories and concepts are universally valid [39]. This kind of research would verify possible similarities among the findings and provide evidence-based decision-making for public policies [40] during a pandemic to promote PA [41,42]. Moreover, this could aid in the establishment and implementation of international suitable policies that could be reached through information sharing. Therefore, in the current multicentric study we aimed to assess the changes in the level of PEx, pain, and psychological impact (i.e., anxiety and stress) before and during the COVID-19 outbreak by using the questionnaire “Physical exercise level before and during social isolation” (PEF-COVID19) in six different countries: Brazil, France, Germany, Italy, Portugal, and Spain. We hypothesized that the COVID-19 lockdown would result in less PEx practice and habits and increased levels of self-reported pain, anxiety, and stress.

## 2. Materials and Methods

### 2.1. Ethics Consideration

This is an observational cross-sectional study through an online self-administered questionnaire survey. The survey was delivered through the internet (email, social media, and social networks). The data collection occurred from 21 April 2020 up to 10 May 2020. The Ethics Committee of the *Hospital Universitário Pedro Ernesto* (HUPE), *Universidade do Estado do Rio de Janeiro* (UERJ), Plataforma Brasil, approved this work with the number CAAE 30649620.1.0000.5259.

The survey was conducted in Brazil, Italy, France, Germany, Portugal, and Spain using translated versions (Italian, French, German, and Spanish) from the original Portuguese version. The study involved researchers from the following institutions: *Universidade do Rio de Janeiro* (Rio de Janeiro, Brazil) and *Universidade do Estado de Santa Catarina* (Florianópolis, Brazil), *Université de Reims* (Reims, France), *Istituto Auxologico Italiano, IRCCS* (Milan and Verbania, Italy), *Leuphana Universität Lüneburg* (Lüneburg, Germany), *Universidade Fenando Pessoa* (Porto, Portugal) and *Universidad de Sevilla* (Spain).

The number of individuals in each country was assessed on 17 May 2020 through a specific ID link created by Google forms for each country.

### 2.2. Sample

A nonrandom convenience sampling of the general population of the six countries, aged more than 18 years old, was invited to participate in this study through a message with a link to the survey. The study’s coordinators distributed the survey by social media, university web pages, and personal contacts, kindly asking them to answer and to distribute the survey to their contacts. The coordination group (Brazil) sent the partners the sampling strategies aimed to achieve different country regions as balanced as possible, contacting colleagues from diverse country regions and asking them to distribute as previously described. The participants agreed to a brief informed consent at the beginning of the survey and at any time, the participants could give up concluding the survey without any penalty or constraints, as stated by the Declaration of Helsinki that contains the ethical principles for medical research involving human subjects.

### 2.3. Survey

The questionnaire “Physical exercise level before and during social isolation” (PEF-COVID19) is aimed at assessing the impact of social isolation due to the COVID-19 pandemic on the levels of PEx and physical and psychological factors in the general population in Brazil, Italy, France, Germany, Portugal, and Spain. For this survey development, a seven-step scale design proposed by Artino et al. [43] was followed. First of all, a literature review was conducted to search for an instrument able to investigate Pex before and during a pandemic. Since this instrument was not available, focus groups with doctors, specialists in the field of the human movement were created. Thereafter, a synthesis of the literature and extensive discussion about the topic were organized to develop the questionnaire to be used. First, the validation was conducted by a panel of experts. Second, interviews were carried out with a few respondents to verify if they were able to understand what was proposed. Third, pilot testing was performed with the target population. The validity (content, construct, clarity, and relevance) was evaluated by a panel of 25 experts (doctors from the health area, specialists in the field of PEx) and the test–retest reliability of this instrument was calculated with a sample of 34 respondents from the general Brazilian population, whose retest was applied 15 days after the first answer. The general validation indexes were all above 0.84 and the test–retest Intraclass Correlation coefficient and Kappa Coefficient were 0.89 and 0.88, respectively [44].

The original instrument was developed in Brazilian Portuguese and after the instrument validation, the questionnaire was translated into Italian, French, German, Portuguese from Portugal, and Spanish, with back-translation to Portuguese to verify possible mistakes.

The instrument has 47 questions with open-ended, closed-ended, Likert scale type of questions and was divided into four sections: (I) subjects’ characterization with demographic, anthropometric, and health status questions (18 items); (II) physical exercise, pain, anxiety, and stress before COVID-19 (13 items); (III) confinement situation update (3 items); (IV) physical exercise, pain, anxiety, and stress during COVID-19 (13 items) [44].

The questionnaire was prepared in the Google Forms platform, hosted via a unique URL, and password-protected to ensure database security [45]. The survey was conducted anonymously.

### 2.4. Data Collection

The questionnaire was distributed using social and network media (WhatsApp, Facebook, Messenger, Instagram, and Linkedin) and email, inviting the target population (aged ≥18 years old) to participate.

### 2.5. Statistical Analysis

Participants’ responses were secured where the data were recorded, resized, and scored on electronic sheets by customized Excel formulas for later statistical analysis.

The statistical analyses were performed using IBM SPSS Statistics for Windows (version 21.0., IBM Corp., Armonk, NY, USA). Descriptive statistical analyses were performed for all variables, which were characterized as *n* (%) or mean ± SD.

Comparisons between the groups for the categorical variables of pain, anxiety, stress, sex, smoking habits, PEx practice, and exercise category were made using Chi-squared analysis. Age distributions, mass, height, body mass index, years of smoking, number of cigarettes per day between the groups were compared using an independent T-test. Post hoc analyses were performed on multiple category data with a Bonferroni correction.

A multivariate logistic regression model was used to predict the nonpractice of PEx during the confinement period for the overall sample, with countries, gender, smoking, age, PEx, PEx practice level before quarantine, and isolation as predictors. Adjusted odds ratios (ORs) and 95% confidence intervals (CIs) were estimated. The overall accuracy of each model was assessed by the area under the curve (AUC). The AUC was determined through receiver operating characteristics (ROC) curve analysis. Statistical significance was set at *p* ≤ 0.05 for all analyses.

## 3. Results

Considering all the respondents, 3335 participants filled the survey. Before data analysis, 141 participants were excluded (4.2%) from the initial respondents because they were from different countries or with ages below 18 years old, thus leaving a total of 3194 questionnaires to be analyzed. The included answers per country and the respective correspondence per millions of inhabitants are indicated in Table 1.

### 3.1. Sociodemographic Characteristics and Sample Health Condition

Table 2 summarizes the sociodemographic characteristics of the sample, divided by country. Considering the mean ages, Italy had the oldest (*p* < 0.001) and Portugal the youngest (*p* < 0.001) sample. The majority (51.8%) of respondents were young adults (from 25 to 44 years). Portugal presented the youngest sample (X^2^(24) = 418.311; *p* < 0.0001). The prevalence of females over males was present in all the country samples, except for Spain having ~60% of male respondents (X^2^(18) = 99.564; *p* < 0.0001). The mean Body Mass Index was on the borderline of overweight (from 25 to 29.9 kg/m^2^) for the Brazilian sample and normal (from 18.5 to 24.9 kg/m^2^) for the other countries. In the whole study group, ~55% were single and ~40% were married, with significant differences between countries (78.9% not married in Portugal) probably related to the marked differences in mean ages (26.6 years in Portugal). Brazil had respondents with a higher education level (masters or doctorate) (X^2^(30) = 1323.534; *p* < 0.0001). The majority of the respondents had university professions (health and social sciences), while in Portugal and Germany the respondents were mainly students (X^2^(24) = 677.326; *p* < 0.0001).

Table 3 summarizes the current health condition of the sample, medication use, and smoking habits. For Brazil, Portugal, and Germany, the most prevalent disease group was that affecting the respiratory system. Italy had the greatest prevalence of cardiac diseases and related conditions and also the oldest sample.

Approximately 40% of Brazilian and Italian respondents used some medication, while the percentages were approximately halved (20%) in the samples from the other countries. The most used medications were for cardiovascular diseases and related conditions in Italy and Brazil and respiratory diseases and allergies in Germany and Spain (in strict relationship with the prevalence of the same diseases).

Considering the overall sample, 8.7% of all respondents smoke; Brazil and Germany presented the lowest percentages. Italy has the highest percentage of smokers (17.6%). The sample from Portugal have smoked for fewer years (*p* < 0.05), however, they have the youngest sample.

### 3.2. Social Isolation Status

**Figure 1:**
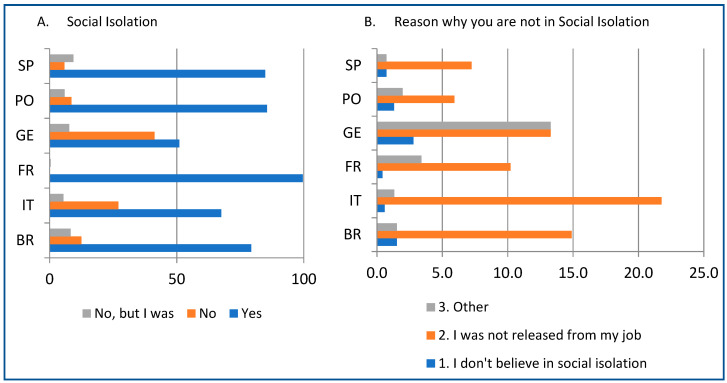
Number of individuals (%) in social isolation (**A**) and reasons why the individual was not isolated (**B**). Sample constituted by general population aged from 18 years old from Brazil (*n* = 1845), Italy (*n* = 680), France (*n* = 236), Germany (*n* = 143), Portugal (*n* = 152), and Spain (*n* = 138). Legend: BR, Brazil; IT, Italy; FR, France; GE, Germany; PO, Portugal; SP, Spain.

As far as the current social isolation status is concerned, Figure 1 shows the percentages of people in social isolation during the survey application and the reasons why they were not in social isolation. The majority of the individuals were in social isolation (~80%); the highest and the lowest rates of the respondents in social isolation were, respectively, 99% in France and 51% in Germany. The subjects that were not in social isolation (12.2%) were not released from their jobs. Few respondents (1.2%) do not believe in social isolation. The mean number of days in social isolation during the survey application were 42.2 ± 11.9 days; Brazil had the fewest days in social isolation (33.5 ± 13.1) and Italy the most (47.8 ± 13.1), followed by Spain (47.1 ± 9.1).

When comparing the overall PEx active groups (in social isolation or not at the moment of the survey application), the percentage of the respondents in social isolation that practiced PEx before the pandemic decreased from 80.4% to 75.4% against 81.1% to 66.9% of the respondents that were not in social isolation during the survey application. Both groups decreased their PEx practice, and no differences were found between them (X^2^(1) = 3.016; *p* = 0.082).

### 3.3. Physical Exercise Practice

Table 4 shows the physical exercise practice before and during the COVID-19 outbreak.

Considering self-related PEx practice before and during the COVID-19 pandemic for the Brazilian sample, the independent Chi-square test showed that the COVID-19 pandemic significantly influenced the PEx self-perception (X^2^(3) = 240.094; *p* < 0.0001) with statistical significance after Bonferroni correction. A greater percentage of “sedentary” and “a bit active” and, by contrast, a lower percentage of “active” and “very active” were found during COVID-19 in comparison with the PEx practice recorded before. The analysis of the European countries showed a significant increase of “sedentary” percentage during COVID-19 and a concomitant reduction of “active” only in Italy (X^2^(3) = 61.890; *p* < 0.0001), while no differences between before and during COVID-19 were detected in France (X^2^(3) = 15.902; *p* < 0.001), Germany (X^2^(3) = 27.708; *p* < 0.001), Portugal (X^2^(3) = 9.483; *p* = 0.024), and Spain (X^2^(3) = 21.609; *p* < 0.001), after Bonferroni correction. 

The general population sample from Brazil and France significantly decreased the PEx practice, (X^2^(1) = 44.254; *p* < 0.0001, X^2^(1) = 10.677; *p* = 0.001, respectively), while no changes were observed in Italy (X^2^(1) = 2.983; *p* = 0.84), Germany (X^2^(1) = 1.059; *p* = 0.3), Portugal (X^2^(1) = 1.363; *p* = 0.24), and Spain (X^2^(1) = 1.529; *p* = 0.21). The number of days of PEx practice for the Brazilian sample during the pandemic also changed (X^2^(3) = 774.122; *p* < 0.0001). During the COVID-19 pandemic, the percentage of Brazilian people performing exercise once a week or every day before COVID-19 significantly increased during COVID-19, while those who performed PEx 2–3 and 4–5 times/week before COVID-19 significantly reduced the frequency. As far as the European countries are concerned, the percentage of people performing PEx 2–3 times/week decreased during COVID-19 in Italy and France, while a significant increase was found in the percentage of people performing PEx 6–7 times/week in these countries (X^2^(3) = 78.083; *p* < 0.0001 and X^2^(3) = 61.947; *p* < 0.0001, respectively). No significant changes of PEx frequency were observed during COVID-19 in Germany (X^2^(3) = 2.214; *p* = 0.52), Portugal (X^2^(3) = 14.137; *p* = 0.003), and Spain (X^2^(3) = 11.265; *p* = 0.01). The duration of the exercise also changed for the Brazilian (X^2^(2) = 480.355; *p* < 0.0001), Italian (X^2^(2) = 179.364; *p* < 0.0001) and Portuguese samples (X^2^(2) = 23.390; *p* < 0.001). Exercise of the short (i.e., 0–30 min) and middle (31–60 min) duration significantly increased during COVID-19 both in Brazil and in Italy, while the long duration (i.e., >61 min) was markedly reduced in these countries and in Portugal. No significant COVID-19-induced changes were found in France (X^2^(2) = 4.457; *p* = 0.1), Germany (X^2^(2) = 20.672; *p* < 0.001), and Spain (X^2^(2) = 18.624; *p* < 0.001).

The PEx type changed for the Brazilian general population (X^2^(3) = 190.663; *p* < 0.0001) comparing the situation before and after the pandemic. A significant increase in the percentage of people that perform aerobic and resistance PEx was detected, this finding being associated with a reduction in the percentage of people performing strengthening PEx. As far as the European countries are concerned no differences were found after the Bonferroni correction in Italy (X^2^(3) = 24.532; *p* < 0.0001), France (X^2^(3) = 13.154; *p* < 0.0001), Germany (X^2^(3) = 12.596; *p* = 0.006), Portugal (X^2^(3) = 36.008; *p* < 0.0001), and Spain (X^2^(3) = 12.800; *p* = 0.005). The fatigue levels were not influenced by COVID-19 pandemic in all samples (Brazil (X^2^(2) = 8.142; *p* = 0.017), Italy (X^2^(2) = 1.801; *p* = 0.4), France (X^2^(2) = 1.119; *p* = 0.5), Germany (X^2^(2) = 1.430; *p* = 0.4), Portugal (X^2^(2) = 0.623; *p* = 0.7), and Spain (X^2^(2) = 2.806; *p* = 0.2)).

The motivation to exercise in terms of performance before and after the pandemic was reduced in the Brazilian (X^2^(3) = 28.111; *p* < 0.0001) and French samples (X^2^(3) =62.420; *p* < 0.0001), while the motivation in terms of health increased only in the Brazilian sample. No changes were found after Bonferroni correction for Italy (X^2^(3) = 5.631; *p* = 0.1), Germany (X^2^(3) = 17.749; *p* < 0.0001), Portugal (X^2^(3) = 3.587; *p* = 0.3), and Spain (X^2^(3) = 2.410; *p* = 0.5).

The physical exercise practice and habits, and self-related fatigue for the overall sample were different when comparing before and during the COVID-19 pandemic.

Considering the period of the day when the exercise was performed before and after the pandemic, significant changes were detected only in the Brazilian (X^2^(3) = 65.373; *p* < 0.0001) and Italian (X^2^(3) = 140.177; *p* < 0.0001) samples. As far as the Brazilian sample is concerned, during COVID-19 a greater percentage of people performed PEx in the afternoon, with a lower percentage during the night. As far as the Italian sample is concerned, the significant increase of people performing PEx in the morning was compensated for by a significant reduction in the afternoon. No significant changes were observed in the other countries (France (X^2^(3) = 19.758; *p* < 0.0001), Germany (X^2^(3) = 5.434; *p* = 0.1), Portugal (X^2^(3) = 19.279; *p* < 0.0001), and Spain (X^2^(3) = 8.100; *p* = 0.4)).

Table 5 presents the results of the multivariate logistic regression model for not practicing of PEx during the social isolation period for the overall sample. The independent variables in the model were: countries, gender, smoking, age (18–24, 25–44, 45–59, >59), PEx, PEx practice level (sedentary, insufficiently, active, very active) and, isolation (yes, no, have been). The multivariate logistic regression model was significant (likelihood-ratio test *p* < 0.000001), with the Nagelkerke R^2^ equal to 29.4% and with the AUC value equal to 0.7876. The categorical variables countries, smoking, PEx, PEx practice level, and social isolation status were *statistically significant (p < 0.05) in at least one subcategory. The variables gender and age were not statistically significant. However, as the age categories increase, the β coefficient also increased and in the last category (more than 60) the β coefficient is 0.368 with p = 0.06.*

### 3.4. Pain and Psychological Impact

Table 6 shows the self-related pain and psychological impact (anxiety and stress) before and during the COVID-19 pandemic.

The presence of self-related pain significantly increased during the COVID-19 pandemic in the Brazilian (X^2^(1) = 33.2; *p* < 0.0001) and Italian samples (X^2^(1) = 16.930; *p* < 0.0001); no significant changes were observed in the other countries (France (X^2^(1) = 2.199; *p* = 0.1), Germany (X^2^(1) = 0.871; *p* = 0.3), Portugal (X^2^(1) = 3.815; *p* = 0.051), and Spain (X^2^(1) = 2.000; *p* = 0.1)). A significant increase of pain (X^2^(4) = 15.215; *p* = 0.004) (localized on the head and neck) was reported only in the Brazilian sample, after the Bonferroni correction (*p* < 0.001). No changes were found for the European countries for body pain between before and during the pandemic, after Bonferroni correction (Italy (X^2^(4) = 2.248; *p* = 0.7), France (X^2^(4) = 25.881; *p* < 0.001), Germany (X^2^(4) = 7.202; *p* = 0.1), Portugal (X^2^(4) = 0.168; *p* = 0.9), and Spain (X^2^(4) = 4.497; *p* = 0.3).

The level of pain changed before and during the COVID-19 pandemic for Brazil (X^2^(2) = 11.635; *p* = 0.003); after the Bonferroni post hoc correction, the level of pain increased in the highest class (i.e., 8–10) (*p* = 0.001). No significant changes in the classes of pain level were observed in the other countries (Italy (X^2^(2) = 5.182; *p* = 0.07), France (X^2^(2) = 13.101; *p* < 0.001), Germany (X^2^(2) = 0.269; *p* = 0.8), Portugal (X^2^(1) = 1.138; *p* = 0.5), and Spain (X^2^(2) = 0.362; *p* = 0.8)) after post hoc correction.

The level of self-related anxiety before and during the COVID-19 pandemic changed in the Brazilian (X^2^(2) = 376.904; *p* < 0.001) and Italian sample (X^2^(2) = 65.852; *p* < 0.001). The percentage of people with a low level of anxiety (i.e., 0–3) significantly was reduced during COVID-19, while the percentage of people with a high level of anxiety (i.e., 8–10) markedly increased after Bonferroni correction in the Brazilian and Italian samples (*p* < 0.008). No significant differences in the level of anxiety was observed in France (X^2^(2) = 13.070; *p* < 0.001), Germany (X^2^(2) = 7.551; *p* = 0.02), Portugal (X^2^(1) = 24.410; *p* < 0.001), and Spain (X^2^(2) = 14.234; *p* = 0.001) after the Bonferroni post hoc correction.

As far as stress is concerned, the independent Chi-square test showed that the COVID-19 pandemic influenced the stress level only in the Brazilian sample (X^2^(2) = 137.857; *p* < 0.001); after the Bonferroni post hoc correction, all stress levels were influenced by the pandemic, with a decrease in the low (0–3) and moderate (4–7) levels and an increase in the highest stress levels (*p* < 0.001). By contrast, after the Bonferroni post hoc correction, no differences in the stress levels were observed in the samples from Italy (X^2^(2) = 6.053; *p* = 0.048), France (X^2^(2) = 2.102; *p* = 0.3), Germany (X^2^(2) = 0.985; *p* = 0.6), Portugal (X^2^(1) = 8.890; *p* = 0.01), and Spain (X^2^(2) = 4.957; *p* = 0.08).

The level of pain and psychological impact for the overall sample were different when comparing before and during the COVID-19 pandemic.

## 4. Discussion

To the best of our knowledge, this study is the first that has investigated the effects of social isolation consequent to the COVID-19 pandemic on PEx practice and habits, self-reported pain, anxiety, and stress levels together in an international multicentric study. As expected, the participants’ lifestyles changed during the quarantine, as they exhibited a lower level of engagement in physical exercise and habits, confirming our hypothesis. Moreover, self-reported pain, anxiety, and stress levels changed among some of the studied countries in different proportions. The findings from this survey showed that the majority of the respondents from Brazil and Europe were in social isolation during the survey application, notwithstanding that the number of days in social isolation was not equal between the countries, probably related to the different epidemic stage in each country. Physical exercise is a powerful tool to be used in the fight to prevent and treat numerous chronic diseases [20], and due to COVID-19, sedentary behavior has been found in the populations of several countries [13,14,30,46,47,48].

It is suggested that the risk of severe disease gradually increases with age starting from around 40 years [49]. Considering the current health condition of the population in the present study, the Brazilian and Italian samples (including older subjects) showed ~40% of concomitant diseases (mainly respiratory and cardiac diseases) requiring specific medications. Among the Brazilian respondents, respiratory diseases were prevalent probably because the majority of the respondents were from big cities (as São Paulo and Rio de Janeiro) where the air pollution may promote adverse effects on the respiratory system [50]. The prevalence of cardiac diseases and related conditions in the Italian sample is probably related to the greater percentage of elderly people. In the other countries, no group of concomitant diseases was markedly prevalent in comparison with the others. Social isolation during the pandemic, although important to slow down the infection rates, contributes to physical inactivity and can cause serious clinical implications for the general population [38,51]. Regular physical activity brings beneficial effects to the general health status [52,53], such as the improvement of physical and physiological health parameters, positive health outcomes, and mental health and well-being [1,54]. In this study, the smoking habit was a predictor for the nonpractice of PEx, and between the participant countries, Italy has the sample with the biggest percentage of smokers. Smoking is a known behavior-related risk factor associated with physical inactivity and obesity to decrease life expectancy [55].

PEx practice and exercise characteristics were investigated by the PEF-COVID19 for before and during the COVID-19 pandemic. The COVID-19 pandemic significantly influenced the PEx self-perception in the Brazilian and Italian samples. This is consistent with recent publications showing a decline in all physical activity levels during home confinement in Brazil and other countries [13,14,34,46,47]. However, no significant differences were detected in the other countries in this study.

According to the multivariate logistic regression model, Germany was the country where the coefficient was significant in the model, with the high practice of PEx. A study on the German population [48] during COVID-19 confinement found that the younger age groups were more likely to maintain PEx compared with older ones. Since the German sample of this survey is predominately composed of young and young-adult, they maintained their self-related PEx practice during the social isolation.

Since many opportunities to be physically active have been suspended in Spain, the time spent in moderate-intensity or vigorous-intensity physical activity during home confinement has been reported to decrease by 92% [30]. By contrast, in this study, the self-related PEx and habits did not change significantly for the Spanish sample; the reason for this difference is probably related to the fact that the Spanish sample in this study was mainly young and young-adult. Additionally, for Portugal, France, and Germany, the sample was younger than for Brazil and Italy. A recent study conducted in a Spanish population (mean age ± SD: 22.7 ± 3.6 years; 57% males) also aimed to determine the effect of confinement on physical activity, sedentary behavior, and other health patterns [30]. The authors observed a significant decline in the levels of physical activity during the lockdown phase. In this study, the independent variables gender and age in the multivariate logistic regression model were not significant in the model.

PEx practice significantly decreased in the Brazilian and French samples, while no changes were observed in the other countries. A study conducted in France and Switzerland showed that the COVID-19 lockdown was associated with lower vigorous physical activity and higher sedentary activity [46] and another one that investigated the Brazilian population found the physical inactivity associated with increased levels of anxiety, depression, and stress during the pandemic [13]. The multivariate logistic regression model shows that the people who did not practice PEx before the pandemic tended to maintain their sedentary behavior. Although the COVID-19 pandemic, as a general rule, has reduced the PEx practice (except for the Portuguese sample, which increased, perhaps as a consequence of the age of the Portuguese sample), it is worth noting that the percentage of subjects still performing PEx practice during the COVID-19 pandemic remained over 65% in all countries. Some governments allowed physical activity practice while others did not; consequently, the measures adopted by the different governments during the quarantine could modulate the results obtained. Moreover, the culture of the practice of physical activity in the different countries contributes to differences in practice rates observed during quarantine [56]. Alternative PEx adaptations have been suggested for the PEx practice during social isolation [41,42,57].

During COVID-19, the weekly frequency for performing PEx in Brazil, Italy, and France changed. These findings seem to suggest that the negative influence of the social isolation consequent to the COVID-19 pandemic mainly affects people performing an intermediate frequency of PEx practice, while people with a low and high frequency increased their amount of weekly PEx practice.

The number of people undertaking exercise of a short (i.e., 0–30 min) and the middle (31–60 min) duration significantly increased during COVID-19 both in Brazil and in Italy, while the long duration (i.e., >61 min) was markedly reduced in these countries and Portugal. In a study conducted in Italy and Spain [34], people also spent less daily time performing physical activity, with only 15% of the individuals achieving at least 60 min of physical activity during the quarantine. A plausible explanation for the reduction of long duration PEx in Italy and Brazil may be related to the higher percentage of people not released from their job in these two countries. Another possible explanation for the overall reduction of PEx duration may be the loss of the possibility to perform outdoor activities related to social isolation (reaching 99% in the French sample). To date, we cannot determine if the reduction of PEx duration during COVID-19 was partially compensated by an increase of the indoor PEx.

The PEx type changed only in the Brazilian sample during the pandemic. A significant increase in the percentage of people performing aerobic and resistance PEx (defined as strength exercise type, usually performed at home with free weights or elastic bands) was detected, this finding being associated with a reduction in the percentage of people performing strengthening PEx (defined as strength exercise type, usually performed in shared places as gyms, with machines or lifting weights), probably due to the forced closing of gyms. However, the closing of gyms was common also to the other European countries, where people did not change the type of PEx performed.

Another exercise characteristic, i.e., the motivation to exercise in terms of performance before and after the pandemic, was reduced in the Brazilian and French samples, while the motivation in terms of health increased only in the Brazilian sample.

Considering the period of the day when exercise was performed before and after the pandemic, significant changes were detected only in the Brazilian and Italian samples. As far as the Brazilian sample is concerned, during COVID-19 a greater percentage of people performed PEx in the afternoon, while a lower percentage during the night. As far as the Italian sample is concerned, the significant increase of people performing PEx in the morning was compensated by a significant reduction in the afternoon.

The presence of pain significantly increased during the COVID-19 pandemic in Brazilian and Italian samples, with no changes being observed in the other countries. A significant increase in pain (localized in the head and neck) was reported only in the Brazilian sample. By contrast, no significant changes in pain level were observed in the other countries, where the population was younger. It is well known that musculoskeletal pain is frequently associated or triggered by psychological stressors [58] or sedentary behaviors [59,60] commonly occurring during social isolation. In fact, during quarantine, the population usually spends a longer time in front of television, computers, or smartphones since any type of activity at home is limited.

Staying at home without the chance to go outside has been suggested to increase mental health problems. The mental health impacts, like anxiety or stress [16,61,62,63] that many will experience in the face of the feeling of fear (fear of the disease, possible loss of family members and friends, economic impact) and isolation from common social life could be a result of the pandemic [33] experienced by a large majority of the population during the COVID-19 pandemic, which may exert negative consequences on the anxiety and stress levels. This survey showed alarming results in some countries (Brazil and Italy), considering the marked increase of anxiety and stress levels during the pandemic, reaching the highest levels (8–10) in Brazil. The greatest percentage of subjects suffering from a medium-high degree of anxiety (40% in Brazil and 30% in Italy) during COVID-19 is explained both by the greater prevalence (occurred in different periods—earlier in Italy, one-month later in Brazil) and morbidity in comparison with the other countries considered in the present study. In Italy, the female gender was associated with increased anxiety, depression, and stress during the pandemic [61]. A study from Brazil showed mild to severe levels of depression during the pandemic and women presented higher levels of anxiety [13]. In line with our observations in Brazil and Italy, a study recently published reported the negative psychological impact of the COVID-19 pandemic in China, where about 50% of respondents faced moderate or severe depression or anxiety symptoms [16]. The negative influence of social isolation due to the COVID-19-related lockdown on psychological well-being (i.e., increased anxiety, depression, and stress) was observed in Italy, with several deaths mainly in the elderly [61].

Considering all these negative behavior changes on the health of several investigated populations during the pandemic [13,14,34,47,48,61,64,65], interestingly, cross-country studies provide local information for how governments can use these data for policymaking; data from population-based surveys and research can be helpful for developing policies and plans to reach high-risk populations demonstrated leadership [66]. At the same time, policies that do not consider cross-country differences may fail, especially in countries with large territories.

Some limitations must be acknowledged. First, the study was conducted on a sample size with markedly different respondents in each of the participating countries. Second, the countries were in different social isolation situations (time in social isolation), and for that reason, the comparisons were not made between countries, to avoid this bias. Nevertheless, this is the first survey aimed to evaluate the combination of physical exercise level, pain, and psychological impact on stress and anxiety levels in different countries during the COVID-19 outbreak. Third, due to the unknown home-quarantine in each country, a specific transcultural translation of the questionnaire was not possible. However, a back-translation to Portuguese was performed to detect some language incongruences. Lastly, although the longitudinal design allows us to evaluate the associations between changes in physical activity, pain, and psychological well-being during the lockdown, because our study was performed over two weeks of the lockdown, there may not have been enough time to determine significant changes in any of the variables.

The strength of this work is related to the characteristics of PEF-COVID19, which is a rapid, objective, feasible, and easy to comprehend questionnaire for the evaluation of changes on the level of PEx, pain, and psychological impact during the social isolation period, which represents useful elements to monitor the quality of life of the general population. With these results public policies must be implemented as home-based exercises for the general population during the confinement; measures with multidisciplinary collaboration must be created for self-management of pain; primary, secondary, and tertiary preventive measures to avoid the increase of the mental health problems are also important; and special care must be given to the elderly to avoid sedentary health style.

## 5. Conclusions

The COVID-19 pandemic has changed the behavior surrounding PEx practice, pain, and mental health of distinct populations. It is expected that with the comparison of some parameters before and during the outbreak, the results regarding this specific sample can aid in the definition or give valuable information about the direction of policies to help the population in the period during and postpandemic. Country of residence, smoking, social isolation, and PEx level were predictors for the nonpractice of PEx.

There is a global concern to collect these data to support decision making and to provide timely interventions, including lifestyle modifications during this time [67]. Consequently, cross-country studies can help with local data to develop public health promotion to maintain a healthy lifestyle before as well as during isolation.

## Figures and Tables

**Table 1:** Number of respondents per millions of people of the countries’ inhabitants.

Country	Inhabitants	Number of Respondents	Respondents Per Million of Person (%)
Brazil *	211,552,573	1845	8.8
Italy ᵜ	60,470,911	680	11.2
France ᵜ	65,258,681	236	3.4
Portugal ᵟ	10,276,617	152	14.7
Germany ᵜ	83,756,401	143	1.7
Spain ᵝ	47,100,396	138	3.1
Overall	478,415,579	3194	6.7

Legend: * IBGE, Instituto Brasileiro de Geografia e Estatística (https://www.ibge.gov.br/apps/populacao/projecao/box_popclock.php); ᵜ https://www.worldometers.info/population/europe/; ᵟ Instituto Nacional de Estatística Português (https://www.ine.pt/xportal/xmain?xpid=INE&xpgid=ine_indicadores&indOcorrCod=0001223&contexto=bd&selTab=tab2&xlang=pt); ᵝ Instituto Nacional de Estadística (INE). (https://www.ine.es/dyngs/INEbase/es/operacion.htm?c=Estadistica_C&cid=1254736176951&menu=ultiDatos&idp=1254735572981). All websites were accessed on 23 May 2020.

**Table 2:** Sociodemographic characteristics of the sample from Brazil (*n* = 1845), Italy (*n* = 680), France (*n* = 236), Germany (*n* = 143), Portugal (*n* = 152) and Spain (*n* = 138), aged from 18 years old (*n* = 3195). Values are presented as mean ± SD or percentage (%).

	Brazil	Italy	France	Germany	Portugal	Spain	Overall
**Age (general)**	39.5 ± 13.3 *	42.9 ± 14.6 *	32.9 ± 10.7	30.4 ± 10.7 *	26.5 ± 7.8 *	33.8 ± 10.8	38.4 ± 13.6
Young (18–24 years)	21.8 ± 1.6 (14.7)	22.6 ± 1.5 (12.3)	21.2 ± 1.8 (29.7)	21.5 ± 1.6 (34)	21.5 ± 1.3 (59.4) ᵜ	22.1 ± 1.5 (17)	21.8 ± 1.6 (18.6)
Young-Adult (25–44 years)	34.7 ± 5.7 (53.3)	33.1 ± 5.8 (38.7)	34 ± 6.4 (52.1)	31.5 ± 4.9 (54.7)	32.8 ± 6.7 (39.3)	32.9 ± 4.7 (72.5)	34 ± 5.8 (50.4)
Adult (45–59 years)	51.8 ± 4.2 (23.4)	52.5 ± 4.1 (35.3) ᵜ	49.5 ± 3.5 (17.4)	51.4 ± 3.5 (9.9)	49.5 ± 3.5 (1.3)	54.6 ± 4.5 (4.2)	51.9 ± 4.2 (22.8)
Elderly (60- years)	65.5 ± 4.6 (8.6)	65 ± 4.6 (13.7)	0 (0)	72.5 ± 4.9 (1.4)	0 (0)	63.3 ± 2.4 (6.3)	65.3 ± 4.6 (8.2)
**Gender**							
Female	70	68.7	55.5	69.9	57.2	38.4 ᵜ	66.7
Male	29.8	30.1	44.5	30.1	42.8	61.6 ᵜ	32.9
Not declared	0.2	1.2	0	0	0	0	0.4
**Anthropometry**							
Mass (kg)	70.9 ± 14.9	70 ± 18.5	69.6 ± 12.7	70.2 ± 11.9	68.4 ± 12.6 ᵟ	72.3 ± 11.9	70.5 ± 15.3
Height (m)	1.67 ± 0.1 *	1.68 ± 0.1 *	1.72 ± 0.1	1.75 ± 0.1 ᵝ	1.7 ± 0.1 *	1.73 ± 0.1	1.68 ± 0.9
BMI (kg/m^2^)	25.3 ± 4.3 *	24.6 ± 5.9 ®	23.4 ± 3.2	22.9 ± 2.8 ᵟ	23.8 ± 4.4	23.9 ± 2.9	24.8 ± 4.6
**Marital Status**							
Not married	38.3 ᵜ	40.1	55.8	64.6	78.9 ᵜ	50	43.6
Married / in a stable relationship	50.8	51.5	36.6	34.7	17.1 ᵜ	49.3	47.6
Divorced	9.4	6.2	7.2	0.7	3.3	0.7	7.5
Widowed	1.5	2.2	0.4	0	0.7	0	1.4
**Education level**							
Elementary school	0.4	0.3	0	0	0	0	0.3
Secondary school	0	9.4 ᵜ	10.2	2.8	0	12.3	3.4
High school	7.9 ᵜ	33.1 ᵜ	79.6 ᵜ	40.5	18.4	0	20.2
Undergraduate	35	40	5.9 ᵜ	18.2	65.8 ᵜ	59.4	35.5
Graduation (masters, doctorate)	56.7 ᵜ	17.2 ᵜ	4.3 ᵜ	38.5	15.8	28.3	40.5
**Profession**							
University profession	63.9	58	61.3	46.7	34.7 ᵜ	78.1	60.9
Nonuniversity profession	7.8 ᵜ	30.4 ᵜ	38.3 ᵜ	5.2	5.3	4.4	14.5
Retired	7.3	4.4	0	0.7	0	0.7	5.2
Student	16.8	7.2 ᵜ	0.4 ᵜ	47.4 ᵜ	60 ᵜ	16.8	16.9
Military	4.2 ᵜ	0	0	0	0	0	2.4
**Working Status**							
Student	15.5 ᵜ	7.5 ᵜ	46.4 ᵜ	50.3 ᵜ	67.1 ᵜ	16.7	20.1
Public sector	30	20.7	13.6	34.3	10.5	24.6	25.9
Private sector	28.3	40 ᵜ	24.7	7	12.5	30.4	28.9
Unemployed	6.5	7.9	3.8	2.1	4.6	6.5	6.3
Retired	7	7.9	0.4	1.4	0	2.2	5.9
SR/U	12.7	16	11.1	4.9	5.3	19.6	12.9

Legend: BMI, body mass index; SR/U, Salary reduction or unemployed due to COVID-19; T-test for independent samples, * when all countries are different between themselves; ^®^ difference of France and Germany; ᵝ difference of France; ᵟ difference of Spain *p* < 0.05. ᵜ Chi-square test for categorical variables, comparisons between countries, with Bonferroni correction *p* < 0.05.

**Table 3:** Current health conditions of the sample from Brazil (*n* = 1845), Italy (*n* = 680), France (*n* = 236), Germany (*n* = 143), Portugal (*n* = 152) and Spain (*n* = 138), aged from 18 years old (*n* = 2860). Values are presented as percentage (%).

	Brazil	Italy	France	Germany	Portugal	Spain	Overall
**Current main disease**							
CD&RC	10.2	17.8	4.3	2.8	3.9	2.2	10.4
ND	0.6	0.6	1.3	1.4	0	0.7	0.7
RD	14	5.1	4.7	9.1	9.9	5.1	10.6
MD	10.7	7.5	6.0	4.9	5.9	8.7	9.1
MH	8.4	3.5	5.1	4.9	7.2	2.2	6.6
No disease	56	65.4	78.7	76.9	73	81.2	62.5
**Medication**							
Yes/No (%)	38/62	38.2/61.8	18.7/81.3	23.1/76.9	18.4/81.6	18.8/81.2	34.2/65.8
Medication type*							
CD&RC/C2+	6.9/6.3	6.2/1.5	2.1/0	1.4/0	0/0	2.2/0	5.6/4
ND/C2+	0.3/0.5	0.3/0.1	0/0	0.7/0	0/0	0/0	0.3/0.3
RD&A/C2+	2.1/2.1	1.9/0.6	5.1/0	7/0	3.3/2	7.2/0	2.8/1.4
MD/C2+	0.8/1.6	1.9/0.3	1.7/0	1.4/0	0/0.7	1.4/0	1.1/1
MH/C2+	5.1/3.1	1.8/0.6	2.5/0	1.4/0	1.3/2	0/0	3.6/2
TH/C2+	3.5/2.8	2.8/1.2	1.7/0	6.3/0	2.7/0	5.1/0	3.4/1.9
CP/C2+	3.7/1.3	4/1.3	1.7/0	2.1/0	4.7/2	2.2/0	3.5/1.1
Food supplements/C2+	1.7/2.5	0.7/0	0/0	0/0	0/0.7	0/0	1.1/1.5
Other/C2+	1.8/3.5	0.6/0.6	3.8/0	0.7/0	2.7/1.3	2.2/0	1.7/2.2
**Smoking**							
Yes(%)/years smoking	4.1 ᵜ/18.6 ± 11.6	17.6 ᵜ/17.3 ± 12	15.7/15 ± 9	2.8/13.5 ± 11.6	16.4/8.9 ± 6 ᵝ	12.3/19 ± 10.8	8.7/16.5 ± 10.9
*n*. cigarettes per day(mean)	10 ± 9.9	9.7 ± 8.7	9.9 ± 6.7	4.1 ± 4.1	8.4 ± 5.2	8.6 ± 6	9.5 ± 6.7
No	95.9	82.4	84.3	97.2	83.6	87.7	91.3

Legend: CD&RC, cardiovascular diseases and related conditions, * antidiabetic, hypocholesterolemic, antihypertensive, anticoagulants and beta-blockers; C2+, combined with two or more medicaments from any category; ND, neurological diseases, * antiepileptic; RD&A, respiratory diseases and allergy, * antihistaminic and antiasthmatic; MD, musculoskeletal, * corticosteroids, antiosteoporosis, nonsteroidal antiinflammatory, painkillers; MH, mental health, * psychotropic; TH, thyroid hormones; CP, contraception, * contraceptive pill; Other, * antiacid, antitumoral, others. ᵜ Chi-square test (*p* < 0.05) for categorical variables, comparisons between countries, with Bonferroni correction. T test for independent samples, ᵝ Portugal was different from Brazil, Italy, France and Spain.

**Table 4:** Physical exercise practice and habits, and self-related fatigue of the sample from Brazil (*n* = 1845), Italy (*n* = 680), France (*n* = 236), Germany (*n* = 143), Portugal (*n* = 152) and Spain (*n* = 138), aged from 18 years old (*n* = 3194), before and during the COVID-19 pandemic. Values are presented as percentages (%).

	PEx Practice before COVID-19							PEx Practice during COVID-19							
	Brazil	Italy	France	Germany	Portugal	Spain	Overall	Brazil	Italy	France	Germany	Portugal	Spain	Overall	
SR Level of PEx															
Sedentary	14.8	11.9	3.8	2.8	12.5	2.9	12.2	28.9 ᵜ	25.6 ᵜ	11.5	8.4	22.4	13	25 ᵜ	
A bit active	24.4	35.6	32.8	14.7	34.2	18.1	27.1	33.2 ᵜ	39.8	28.1	32.9	28.3	32.6	34 ᵜ	
Active	43.7	43.7	47.7	52.4	31.6	52.2	44.2	32 ᵜ	27.5 ᵜ	37.4	48.2	36.8	39.2	32.7 ᵜ	
Very active	17.1	8.8	15.7	30.1	21.7	26.8	16.5	5.9 ᵜ	7.1	23	10.5	12.5	15.2	8.3 ᵜ	
PEx Practice															
Yes	80.4	72.8	92.8	95.8	70.4	92.8	80.5	71	68.5	82.6	93	76.3	88.4	73.4 ᵜ	
No	19.6	27.2	7.2	4.2	29.6	7.2	19.5	29 ᵜ	31.5	17.4 ᵜ	7	23.7	11.6	26.6 ᵜ	
Frequency															
1 t/w	4.7	13	18.7	3.6	8.9	3.1	7.6	9.1 ᵜ	10.7	6.9	5.2	9.6	4.1	8.9 ᵜ	
2–3	45.6	57.5	52.3	30.5	46.4	34.9	47.5	40.6 ᵜ	35.4 ᵜ	30.7 ᵜ	35.8	27.8	23.6	37.2 ᵜ	
4–5	33.2	23	20.9	44.9	35.8	43.4	31.2	32.2 ᵜ	30.3	30.3	36.6	37.4	35.8	32.3 ᵜ	
6–7	16.5	6.5	8.1	21	8.9	18.6	13.7	18.1 ᵜ	23.6 ᵜ	32.1 ᵜ	22.4	25.2	36.5	21.6 ᵜ	
Duration															
0–30 min	5.6	8.4	11.1	3.7	5.6	3.1	6.4	30.4 ᵜ	38.1 ᵜ	15.6	15.1	22.6	11.6	29.1 ᵜ	
31–60	14.4	14.4	20.8	17.6	17	12.5	15.1	28 ᵜ	26.2 ᵜ	25.7	31.1	30.4	28.1	27.7 ᵜ	
61-	80	77.2	68.1	78.7	77.4	84.4	78.5	41.6 ᵜ	35.7 ᵜ	58.7	53.8	47 ᵜ	60.3	43.2 ᵜ	
PEx type															
Aerobic	44.4	77.8	73.1	51.1	37.6	39.4	53.4	54.9 ᵜ	74.2	56.9	70.1	59.3	27.9	58.9 ᵜ	
Resistance ‡	10.3	4.7	20.1	2.9	15.6	13.4	9.9	21.2 ᵜ	12.7	31.2	3.8	30.1	29.5	20.1 ᵜ	
Strengthening ‡‡	28.9	4.7	6.8	12.4	31.2	25.2	21.3	11.6 ᵜ	5.5	11.9	9.7	7.1	16.4	10.2 ᵜ	
Other/CM	16.4	12.8	0	33.6	15.6	22	15.4	12.3	7.6	0	16.4	3.5	26.2	10.8 ᵜ	
SR Fatigue PEx															
0–3	28.6	21.9	21.8	17.9	12.5	3.9	24.4	24.2	24.2	25.7	23.7	15.7	9	23.2 ᵜ	
4–7	50.8	64.1	70.9	62.1	71.4	65.4	57.2	52.9	59.9	68.3	57.8	67	63.9	56.9 ᵜ	
8–10	20.6	14	7.3	20	16.1	30.7	18.4	22.9	15.9	6	18.5	17.3	27.1	19.9 ᵜ	
Motivation															
Entertainment	7.8	22.8	7.7	18.1	21.4	11	12.2	8.2	28.7	15.1	6.7	23.5	11.4	13.8 ᵜ	
Performance	10	11	31.9	24.6	15.2	17.3	13.1	5.6 ᵜ	8.2	3.7 ᵜ	14.2	7.8	12.2	6.8 ᵜ	
Aesthetics	9.8	20.5	32.8	10.9	17	3.9	13.9	7.1	20.1	40.4	9	14.8	7.3	12.8 ᵜ	
Health	72.4	45.7	27.6	46.4	46.4	67.8	60.8	79.1 ᵜ	43	40.8	70.1	53.9	69.1	66.6 ᵜ	
Period PEx															
Dawn	1.5	10.5	3.8	5.8	0.9	6.2	4	0.7	14.1	2.3	9	0.9	4.1	4.2 ᵜ	
Morning	45.7	30.4	20.4	16.7	22.5	27.2	37.4	42.5	73.7 ᵜ	30.7	25.6	16.5	25.2	45.6 ᵜ	
Afternoon	19.1	53.8	57.4	68.8	57.7	55	35	31.5 ᵜ	9.4 ᵜ	61	60.2	80	67.5	34.1 ᵜ	
Night	33.7	5.3	18.2	8.7	18.9	11.6	23.7	25.3 ᵜ	2.8	6	5.2	2.6	3.2	16.2 ᵜ	

Legend: PEx, Physical Exercise; TS, total sample; SR, self-related; *t*/*w*, times/week; min, minutes; CM, combined modalities; ‡ for strength exercise types: free weights and elastic bands; ‡‡ for strength exercise types: weight training with machines or lifting weights; ᵜ Chi-square test (*p* < 0.05) with Bonferroni correction for categorical variables, comparisons intracountries, before and during COVID-19.

**Table 5:** Results of the multivariate logistic regression for the nonpractice of PEx during the quarantine period for the overall sample (*n* = 3195).

Data File	β	*S.E.*	*z* Value	*df*	*p*	*OR*	95% CI *OR*
Brazil	0.335	0.248	1.352	1709	0.176	1.398	[0.859, 2.270]
Italy	0.180	0.255	0.707	644	0.480	1.197	[0.726, 1.970]
Portugal	−0.029	0.303	−0.097	150	0.923	0.971	[0.533, 1.751]
Germany	−1.010	0.419	−2.413	141	0.016 *	0.364	[0.153, 0.801]
Spain	−0.417	0.369	−1.131	137	0.258	0.659	[0.313, 1.336]
France	−0.008	0.286	−0.028	232	0.978	0.992	[0.563, 1.730]
Gender (male)	0.088	0.101	0.867	1002	0.386	1.091	[0.895, 1.330]
Gender (prefer not to say)	0.804	0.722	1.112	10	0.266	2.234	[0.509, 9.007]
Smoking (no)	−0.314	0.159	−1.971	2748	0.049 *	0.73	[0.536, 1.001]
Age (2544)	0.211	0.141	1.493	1519	0.135	1.235	[0.939, 1.634]
Age (45–59)	0.247	0.160	1.548	689	0.122	1.28	[0.938, 1.754]
Age (more than 60)	0.368	0.200	1.841	247	0.066	1.445	[0.976, 2.139]
PEx practice (no)	0.706	0.126	5.584	585	<0.001 *	2.026	[1.581, 2.596]
PEx level (insuf. active)	−1.017	0.146	−6.972	807	<0.001 *	0.362	[0.271, 0.481]
PEx level (active)	−2.035	0.162	−12.571	1334	<0.001 *	0.131	[0.095, 0.179]
PEx level (very active)	−2.720	0.223	−12.206	503	<0.001 *	0.066	[0.042, 0.101]
Social isolation (no)	0.590	0.132	4.471	455	<0.001 *	1.805	[1.392, 2.336]
Social isolation (have been)	0.449	0.180	2.499	208	0.012 *	1.566	[1.097, 2.219]

Legend: β = regression coefficient; *df* = degrees of freedom; *OR* = odds ratio; CI = confidence interval. * *p* < 0.05.

**Table 6:** Level of pain and psychological impact of the sample from Brazil (*n* = 1845), Italy (*n* = 680), France (*n* = 236), Germany (*n* = 143), Portugal (*n* = 152) and Spain (*n* = 138), aged from 18 years old (*n* = 3194), before and during the COVID-19 pandemic. Values are presented as percentages (%).

	Pain, Anxiety, and Stress Before COVID-19							Pain, Anxiety, and Stress during COVID-19						
	Brazil	Italy	France	Germany	Portugal	Spain	Overall	Brazil	Italy	France	Germany	Portugal	Spain	Overall
Pain														
Yes	34.6	33.5	41.7	24.5	27.6	31.9	34	44 ᵜ	44.4 ᵜ	35	29.4	38.2	40.6	42.3 ᵜ
No	65.4	66.5	58.3	75.5	72.4	68.1	66	56	55.6	65	70.6	61.8	59.4	57.7 ᵜ
Pain BR ^‡^														
H&*n*	4.1	10.2	7	8.5	3.9	4.5	5.8	8.2 ᵜ	13.8	7.5	6.8	5.4	5.7	9 ᵜ
UL	4.6	6.8	7.7	6.6	5.1	5.2	5.4	6.3	10.1	3.8	4.1	8	9.3	7 ᵜ
Back	15.8	11.7	10.6	0.9	11.6	9.8	13.4	18.4	13.6	15.4	8.2	16.1	16.4	16.5 ᵜ
Hip	2.6	1.6	7.7	1.9	0.6	2.6	2.6	2.7	1.6	0	2.1	0.7	0.7	2.1 ᵜ
LL	7.4	3.2	8.8	6.6	6.4	9.8	6.6	8.4	5.3	8.3	8.2	8	8.5	7.7 ᵜ
Level of pain ^≈^														
0–3	33.6	22.8	14.6	33.3	39.5	21.2	29.6	28.7	22.2	31	36.4	29.8	21.2	27.6 ᵜ
4–7	55.6	66.8	72.5	55.6	51.2	59.6	59.2	55.1	60.6	52.4	50	61.4	63.6	56.5 ᵜ
8–10	10.8	10.4	12.9	11.1	9.3	19.2	11.2	16.2 ᵜ	17.2	16.6	13.6	8.8	15.2	15.9 ᵜ
Level of Anxiety ^≈^														
0–3	42.8	60.6	56.6	78.4	59.8	55.1	50.6	21.6 ᵜ	40.9 ᵜ	41.3	63.7	38.2	32.6	30.4 ᵜ
4–7	47.3	33.6	35.8	19.5	37.5	36.3	41.3	44.3	43	44.6	32.2	44.7	52.9	43.9 ᵜ
8–10	9.9	5.8	7.6	2.1	2.7	8.6	8.1	34.1 ᵜ	16.1 ᵜ	14.1	4.1	17.1	14.5	25.8 ᵜ
Level of Stress ^≈^														
0–3	34.1	34.3	48.1	36.4	46.7	27.5	35.6	26.7 ᵜ	34.8	42.6	41.3	36.8	39.9	31.3 ᵜ
4–7	52	49.9	42.1	45.5	46.7	50.7	50.2	43.4 ᵜ	44.7	44.3	39.9	46.1	44.1	43.7 ᵜ
8–10	13.9	15.8	9.8	18.1	6.6	21.8	14.2	29.9 ᵜ	20.5	13.1	18.8	17.1	16	25 ᵜ

Legend: PE, physical Exercise; BR, body regions; ^‡^ values are relative to the respondents with pain; H&*n*, head and neck; UL, upper limbs; LL, lower limbs. ^≈^ values are relative to the given answers (100%); ᵜ Chi-square test (*p* < 0.05) with Bonferroni correction for categorical variables, comparisons intracountries, before and after COVID-19.

## Data Availability

Data available on request due to restrictions eg privacy or ethical. The data presented in this study are available on request from the corresponding author.
